# Optimization of passive micromixers: effects of pillar configuration and gaps on mixing efficiency

**DOI:** 10.1038/s41598-024-66664-z

**Published:** 2024-07-15

**Authors:** Ali Kheirkhah Barzoki

**Affiliations:** Department of Mechanical Engineering, Sharif University of Technology, Tehran, Iran

**Keywords:** Micromixer, Mixing, Pillar, Obstacle, Mass transfer, Microfluidic, FEM, Mechanical engineering, Fluid dynamics

## Abstract

Chemical bioreactions play a significant role in many of the microfluidic devices, and their applications in biomedical science have seen substantial growth. Given that effective mixing is vital for initiating biochemical reactions in many applications, micromixers have become increasingly prevalent for high-throughput assays. In this research, a numerical study using the finite element method was conducted to examine the fluid flow and mass transfer characteristics in novel micromixers featuring an array of pillars. The study utilized two-dimensional geometries. The impact of pillar configuration on mixing performance was evaluated using concentration distribution and mixing index as key metrics. The study explores the effects of pillar array design on mixing performance and pressure drop, drawing from principles such as contraction–expansion and split-recombine. Two configurations of pillar arrays, slanted and arrowhead, are introduced, each undergoing investigation regarding parameters such as pillar diameter, gap size between pillar groups, distance between pillars, and vertical shift in pillar groups. Subsequently, optimal micromixers are identified, exhibiting mixing efficiency exceeding 99.7% at moderate Reynolds number (Re = 1), a level typically challenging for micromixers to attain high mixing efficiency. Notably, the pressure drop remains low at 1102 Pa. Furthermore, the variations in mixing index over time and across different positions along the channel are examined. Both configurations demonstrate short mixing lengths and times. At a distance of 4300 μm from the inlet, the slanted and arrowhead configurations yielded mixing indices of 97.2% and 98.9%, respectively. The micromixers could provide a mixing index of 99.5% at the channel’s end within 8 s. Additionally, both configurations exceeded 90% mixing indices by the 3 s. The combination of rapid mixing, low pressure drop, and short mixing length positions the novel micromixers as highly promising for microfluidic applications.

## Introduction

The concept behind microfluidic devices is to downsize a traditional laboratory into a miniaturized chip, incorporating various functions such as detection^1,2^, mixing^3,4^, synthesis^5–8^, and separation^9–11^ at the micro- and nano-scale. These devices are extensively used in chemical analysis^12,13^, chemical synthesis^14,15^, and biomedical analysis^16^ due to their speed, accuracy, and minimal reagent requirement. Efficient mixing holds significant importance in these devices, making the attainment of quick and uniform mixing with minimal fabrication complexity. Extensive research conducted over time has resulted in the introduction of diverse micromixer designs.

Microfluidic flow in microchannels is typically laminar due to the small length scale, characterized by a low Reynolds number (Re). In the co-flow of two homogeneous and miscible fluids within a channel, mass transport is mainly governed by molecular diffusion. This makes microfluidic mixing particularly challenging. This situation exacerbates in applications where the mixing involves large analytes such as DNA or nanoparticles. One approach to microfluidic mixing involves the use of droplets to mix reagents in the process of droplet formation. When two immiscible fluids meet at a junction, various forces, such as viscosity, surface tension, and pressure gradient, come into play, causing the droplet to separate from the dispersed phase and flow downstream. By maintaining a controlled droplet size, this method prevents axial dispersion of mixing components, allowing for swift mixing through the internal circulation of the droplet^17,18^. Kheirkhah Barzoki introduced three novel droplet generators that significantly enhance mixing efficiency to approximately 85%, all while maintaining smaller droplet sizes compared to typical droplet generators^19^. Belousov et al. introduced an asymmetric flow focusing droplet generator to enhance mixing during the droplet formation stage, demonstrating a six-fold increase in mixing speed compared to a symmetric design^20^. Several studies have concentrated on improving the mixing index in microfluidic systems based on droplets by integrating micromixers immediately following the droplet generator^21–25^. The incorporation of micromixers induces advection within the droplets, consequently enhancing the mixing process. Nonetheless, droplet microfluidics exhibits heightened sensitivity to the conditions governing droplet formation, requires more intricate instrumentation, and introduces certain limitations related to the compatibility of materials with oils and surfactants.

Another promising approach is the utilization of micromixers to enhance the mixing of analytes. In the last two decades, there has been a surge in the design and study of micromixers^4,18,21,25–28^. These micromixers generally fall into two main categories: active mixers, which enhance mixing through external forces that disturb and stir the fluid^26,27,29–35^, and passive mixers, where convective re-circulations and vortices induce folding and extension of the fluid–fluid interface^25,28,36–47^. This results in a reduction in the length-scale minimizing the distance over which molecular diffusion needs to act for complete mixing. Active approaches can achieve high mixing performance but are often more complex and expensive to integrate and fabricate compared to passive mixers. On the other hand, passive mixers can also achieve a high degree of mixing, but they may require significant pressure to drive flow and need a simple structure to facilitate fabrication and prevent channel clogging.

The performance of passive micromixers is inherently influenced by the channel geometry. A common strategy for achieving mixing in a microchannel involves employing a series of repetitive mixing units. Each mixing unit represents a geometric structure specifically designed to elongate the fluid interface through fluid–geometry interaction. Several studies have concentrated on the design of various serpentine channels aimed at inducing secondary flows and augmenting mixing efficiency^22,28,37,48–51^. An alternative strategy for improving mixing efficiency involves incorporating obstacles into the channel^41–46,52–55^. The presence of obstacles in the channel leads to an extended fluid–fluid interface, resulting in increased mixing as the fluids traverse these impediments. In certain studies, researchers have integrated serpentine channel designs and active methods with obstacles, combining the advantages of both approaches^26,36,50,56–62^. Bhattacharya et al. developed T-shaped wavy-walled micromixers with embedded obstacles and demonstrated that incorporating obstacles can significantly improve mixing efficiency^39^. Introducing obstacles into the channel may result in an elevated pressure drop, but it presents the advantage of enabling proper mixing of reagents within a relatively compact length. Conversely, serpentine designs can elongate the channel length and introduce complexity to the overall design. Consequently, devising an optimal and straightforward arrangement of obstacles holds significant promise.

In this study, the mixing of two co-flowing fluids through the inertial flow deformation induced by a series of two-dimensional cylindrical obstacles (pillars) is investigated. Two distinct configurations are examined, manipulating parameters such as pillar diameter, gap size between pillar groups, distance between pillars, and the vertical shift between groups of pillars. Both configurations showed the potential for achieving a mixing index higher than 99.7% within a relatively short channel length and time making them applicable to diverse applications, particularly those requiring a compact design.

## Mathematical model

### Geometry of micromixers

**Figure 1:**
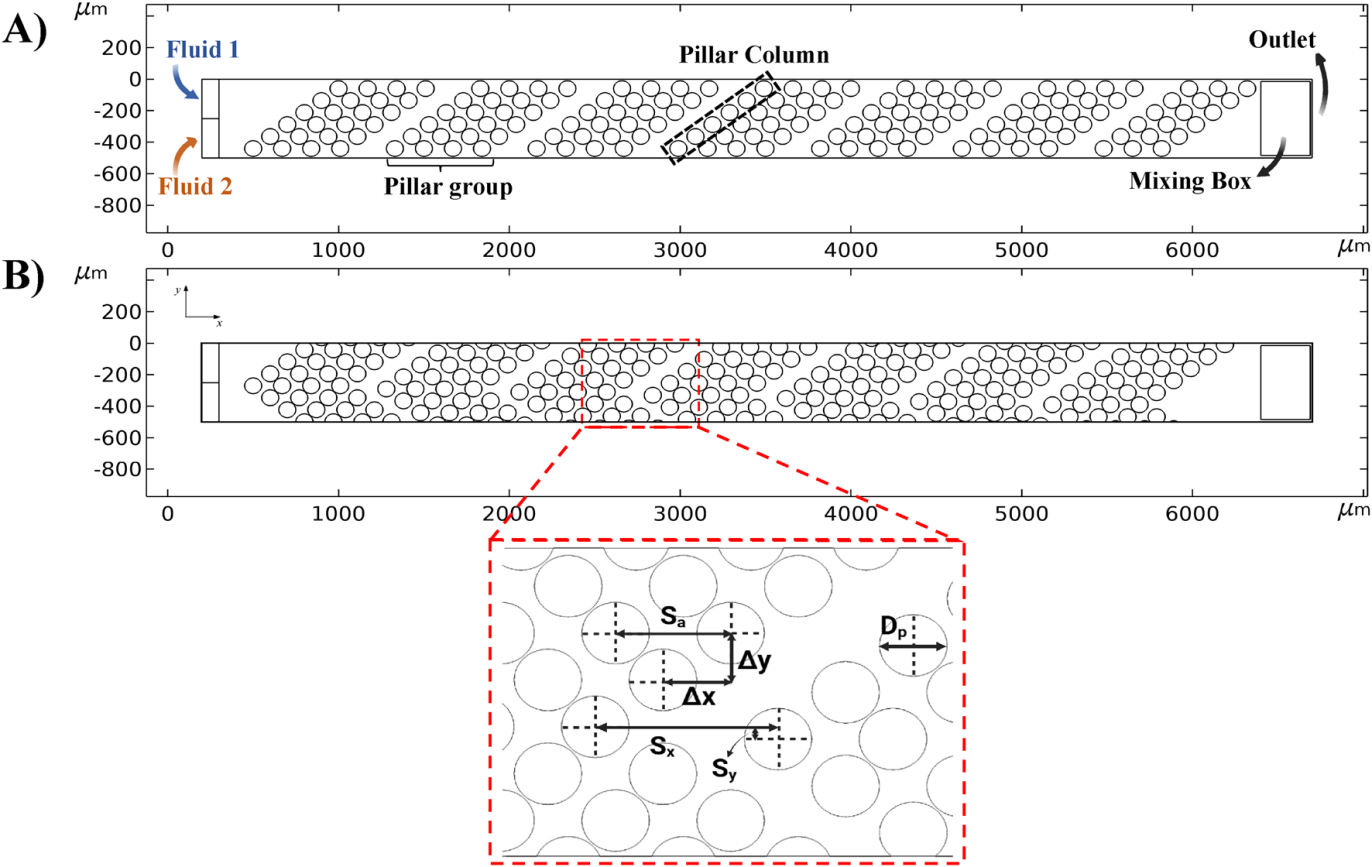
Sketch of the geometries. (**A**) Slanted configuration and flow path, and (**B**) arrowhead configuration and geometrical parameters.

In this research, the microchannel features a straight design with an array of pillars. As depicted in Fig. 1, there are two primary pillar configurations: slanted configuration (Fig. 1A) and arrowhead configuration (Fig. 1B). The system includes two fluid inlets with dye concentrations of 1 and 0 $\text{mol}/{\text{m}}^{3}$. It is important to note that all properties of the two fluids are identical, except for the dye concentration. The channel has an outlet at its end, and a mixing box is utilized at the end of the channel to compute the mixing index within it (Fig. 1A).

In both configurations, there are some groups of pillars, each consisting of four rows (see Fig. 1A). These groups are separated by a specific gap size (G), which is calculated as S_x_ − S_a_ (Fig. 1B). Within each pillar group, the spacing between pillars in each column (Fig. 1A) is determined by two fixed parameters, ∆x and ∆y (Fig. 1B). The parameter S_a_ establishes the distance between the columns of pillars in each group, with its values specified in Table 1. The distance between separate groups of pillars is determined by S_x_ and S_y_. As previously mentioned, S_x_ equals S_a_ + G. If G equals 0, there will be no gap between the groups of pillars, resulting in a continuous array through all the channel. Table 1 provides the geometrical parameters and their values. To be noted that D_p_, S_a_, S_y_, and G get various values and the effect of changing each parameter on the mixing index is studied in this research. Parameters $\Delta x$ and $\Delta y$ have a fixed value in all simulations. The optimization process utilizes the values presented in Table 1 for the other parameters. Subsequently, the micromixers with optimal values for each parameter providing the best mixing index are determined.

**Table 1:** Geometric dimensions of the micromixers.

Parameters	*D*_*p*_	$\Delta x$	$\Delta y$	*S*_*a*_	*S*_*x*_	*S*_*y*_	*G* = *S*_*x*_* − S*_*a*_
Values (μm)	50 75 100 125	100	76	170 190 210 230 250	*S*_*a*_ + *0* *S*_*a*_ + 50 *S*_*a*_ + 100 *S*_*a*_ + 150 *S*_*a*_ + 200	10 20 30 40	0 50 100 150 200

### Governing equations

The mixing analysis involves two key principles: mass transport and momentum transport. To obtain the flow field for incompressible fluids, the conservation of mass (continuity) and the conservation of momentum (Navier–Stokes equations) must be solved. Considering laminar flow ($Re\approx 1$), steady state, and incompressible fluid, these equations are outlined below:

1 $$\nabla \cdot \vec{u} = 0$$

2 $$\rho \left( {\vec{u} \cdot \nabla } \right)\vec{u} = - \nabla P + \mu \nabla^{2} \vec{u}$$

where $\overrightarrow{u}$ denotes the velocity vector ($\text{m}/\text{s}$), $\mu$ represents the dynamic viscosity (${\text{Pa }}\;{\text{s}}$), $P$ shows the pressure ($\text{Pa}$), and $\rho$ denotes the fluid density ($\text{kg}/{\text{m}}^{3}$).

Equation (1) ensures that the fluid density remains constant (i.e., the fluid is incompressible), meaning that the volume of fluid remains constant as it flows. In Eq. (2), the term on the left is the convective acceleration term, representing the change in velocity due to the movement of the fluid in space. The first term on the right represents the pressure gradient force per unit volume, indicating how pressure variations in the fluid drive the flow, causing the fluid to move from regions of high pressure to low pressure. The second term on the right represents the viscous forces per unit volume in the fluid, describing the diffusion of momentum due to viscosity. This term accounts for the internal friction within the fluid, smoothing out velocity differences.

To model the mixing phenomenon, the convective-diffusive mass transport equation was employed:

3 $${{\partial c} \mathord{\left/ {\vphantom {{\partial c} {\partial t}}} \right. \kern-0pt} {\partial t}} + \vec{u} \cdot \nabla c = D\nabla^{2} c$$

Here, D represents the diffusion coefficient (${\text{m}}^{2}/\text{s}$) and c denotes the dye concentration ($\text{mol}/{\text{m}}^{3}$). The first term on the left represents how the concentration (c) changes with time at a given point in space. The second term on the left, the convective term, represents the transport of the substance due to the bulk motion of the fluid, describing how the concentration changes as it is advected by the velocity field. The term on the right represents the spread of the substance due to diffusion, describing the process by which the substance spreads out due to random molecular motion, moving from regions of higher concentration to regions of lower concentration.

To be noted that by considering steady state, the velocity and pressure distribution was obtained. Afterwards, the velocity field was seeded to the mass transport equation (Eq. 3). In this case, it was considered that the dye concentration is diluted and has no effect on the fluid flow. This assumption lowers the run time of the solution, and studying the mass transport after reaching a steady state confirms the validity of this assumption.

To determine the mixing index (MI), the dye concentration in each mesh cell was taken into account, employing the following formula^63^:

4 $$MI~\left( \% \right) = \left( {1 - \sqrt {\frac{{\int {\int {\left( {c - \bar{c}} \right)^{2} } } dA}}{{A~ \cdot ~~\overline{{c~}} ^{2} }}} } \right) \times 100$$

where, c and $\overline{c }$ represent the dye concentration and average dye concentration, respectively. A denotes the area in which the mixing index is calculated. MI varies between 0 and 100%. $MI=0$ corresponds to no mixing, while $MI=100\%$ means complete mixing. As the MI increases, the mixing efficiency is improved, and the concentration of species becomes more uniform. In this study, the mixing index was calculated in a specific area at the end of the channel, called mixing box (see Fig. 1A).

### Boundary conditions

The study on fluid flow in the microchannel implemented the no-slip boundary condition on the channel walls, while maintaining a constant zero gauge pressure at the outlet. For the inlets, constant flow rate (25 $\upmu\text{l}/\text{min}$) with a parabolic velocity distribution was considered. Considering the characteristic length of the inlet, Re was obtained 0.83. Deionized (DI) water, with a density of 1000 $\text{kg}/{\text{m}}^{3}$ and a dynamic viscosity of 0.001 ${\text{Pa }}\;{\text{s}}$, was chosen as the working fluid. To be noted that since the dye solution was considered to be diluted, the density and viscosity of the solution was considered as DI water. For the mass transfer investigation, the walls were subjected to a no-mass flux boundary condition, and constant concentrations of 0 and 1 $\text{mol}/{\text{m}}^{3}$ were set at the inlets. A diffusivity constant of $5.75 \times 10^{ - 10} \;{\text{m}}^{{2}} {\text{/s}}$ was selected ^64^.

### Numerical model

The simulations assume that the fluids (i.e. water and dye solution) are continuous, incompressible, isothermal, and Newtonian with constant physical properties in the laminar regime. The assessment of mixing efficiency, which measures the uniformity of various analytes, was conducted through numerical simulations employing the finite element method (FEM). This process involved the simultaneous solution of two sets of equations. The fluid flow equations were addressed using the Parallel Direct Sparse Solver (PARDISO) with a residual tolerance of ${10}^{-3}$, while the mixing equation was solved using the Multifrontal Massively Parallel Sparse Direct Solver (MUMPS) with left preconditioning and a residual tolerance of 1E−3. First-order and second-order elements were utilized for discretizing pressure and velocity, respectively, and linear discretization was applied to the mixing study. The Newton method was employed for the linearization of non-linear equations at each time step. The determination of the time step (Δτ) relied on the Courant number ($Co=0.25$) to accommodate unsteady simulations (Eq. 5). In this equation, u and $\Delta h$ denote the average fluid velocity and mesh size, respectively. Triangular (tri) elements were employed for discretizing the entire domain, encompassing boundary layers. Although three-dimensional (3D) geometries are generally more precise, 2D geometries were chosen for the simulations due to their ability to provide valuable information and maintain acceptable consistency with experimental data without necessitating extensive computational resources^20,65–67^.

5 $$Co=u\Delta \tau /\Delta h$$

### Mesh independence study

**Figure 2:**
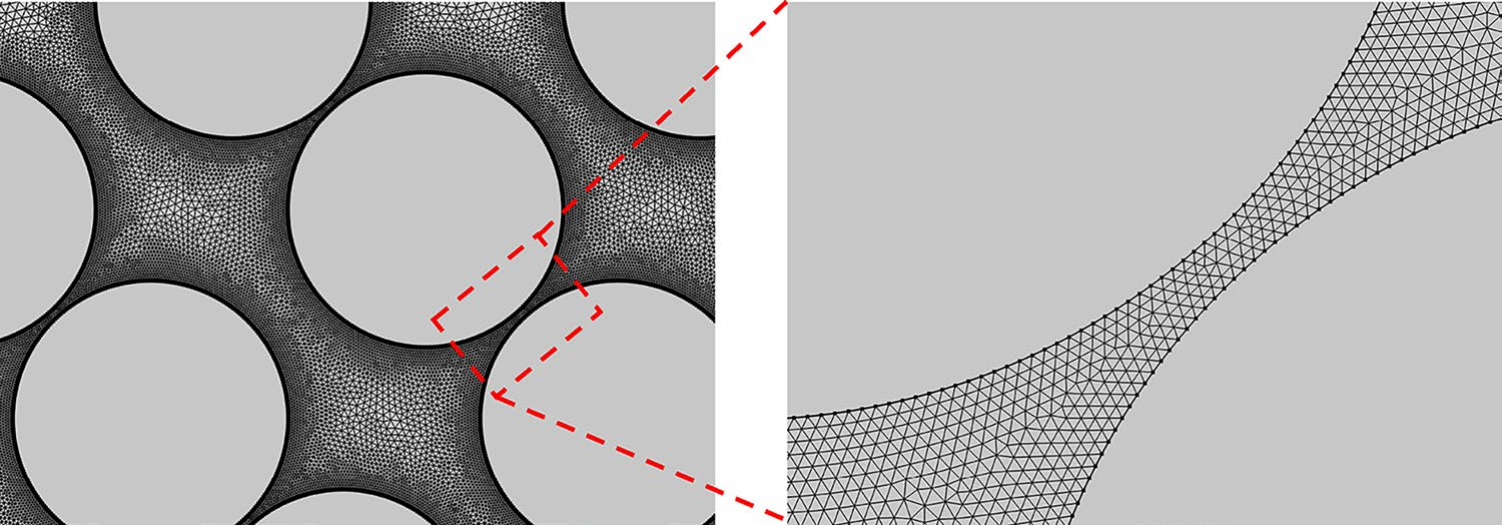
The sample mesh generated in the computational domain.

As previously stated, the study examines various configurations of pillars in a rectangular channel to assess mixing efficiency. To ensure reliable and consistent numerical simulation results, each microchannel must have a designated mesh configuration containing an appropriate number of grids. Consequently, a thorough mesh independence study is conducted for the worst geometry, slanted configuration with $G=0$, $S_{a} = 170 \;\upmu\text{m}$, and $D_{p} = 100 \;\upmu\text{m}$, and the corresponding results are detailed in Table 2. This geometry is the worst geometry because the pillars are tightly packed next to each other and the mesh quality between pillars have to be refined (see Fig. 2). To refine the mesh quality between pillars, a distribution of 320 elements on the boundary of each pillar was considered. The maximum element growth rate, the curvature factor, and the minimum element size were considered 1.2, 0.25, and 0.1 μm, respectively. The outcomes reveal an improvement in the mixing index and pressure drop when the number of elements is increased to 760,998 and 1,216,696. However, the selection of element size of 15 μm with number of elements of 760,998 is favored for its advantages in terms of both reduced computational time and high accuracy. Consequently, the subsequent simulations adopted a mesh element size of 15 μm.

**Table 2:** Mesh independence study.

Number of elements	Pressure drop (Pa)	Mixing index (%)
150,392	9494.3	94.09
240,627	9345.2	89.93
372,562	9201.8	86.51
596,723	9118.7	64.02
760,998	9038.5	63.81
1,216,696	9039.1	63.79

### Model validation

**Figure 3:**
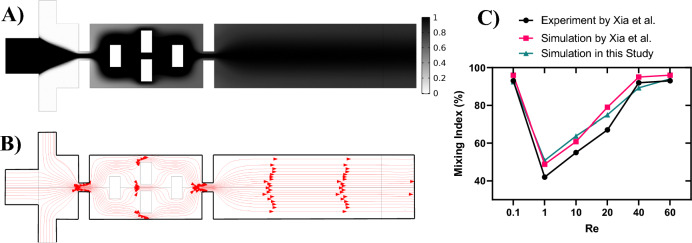
Model validation. (**A**) Dye concentration and (**B**) streamlines at $Re=0.1$ in the geometry of experimental study by Xia et al.^68^. (**C**) Comparison of numerical results of mixing indices with results by Xia et al.^68^.

To validate our computational model, the results from a previous experimental study conducted by Xia et al.^68^ were utilized. This study features obstacles and gaps, as in this study. For this purpose, one of their geometries with suitable boundary conditions and mesh configuration, as shown in Fig. 3A,B, was simulated. Figure 3C illustrates the numerical mixing index results obtained through our computational model, along with the experimental and numerical results by Xia et al.^68^. It is evident that the numerical results surpass the experimental data, attributed to certain assumptions made in the simulation. Across the range of Re = 0.1, 1, 10, 20, 40, and 60, the mixing index progressively increases with Re, demonstrating a consistent correspondence between the mixing index results from our computational model and those reported by Xia et al.^68^.

## Results and discussion

### Effect of S_a_

**Figure 4:**
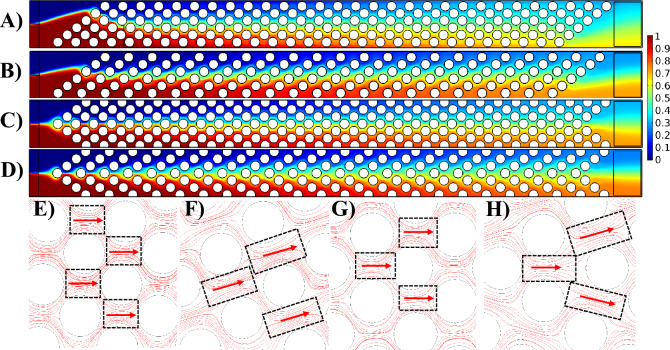
Demonstration of flow pattern and mixing efficiency with respect to S_a_. Dye concentration throughout the channel for the slanted configuration with (**A**) $S_{a} = 190\;\upmu\text{m}$, (**B**) $S_{a} = 250\;\upmu\text{m}$, and arrowhead configuration with (**C**) $S_{a} = 190\;\upmu\text{m}$, and (**D**) $S_{a} = 250\;\upmu\text{m}$. Magnified view of the streamlines passing through the pillars in slanted configuration with (**E**) $S_{a} = 190\;\upmu\text{m}$, (**F**) $S_{a} = 250\;\upmu\text{m}$, and arrowhead configuration with (**G**) $S_{a} = 190\;\upmu\text{m}$, and (**H**) $S_{a} = 250\;\upmu\text{m}$.

In this section, the impact of the S_a_ on both the mixing index and pressure drop is investigated, considering a zero gap size (G) and a pillar diameter of $100 \;\upmu\text{m}$. Figure 4 illustrates the flow pattern and dye concentration within the channel, showcasing the mixing efficiency for various S_a_ values in both slanted and arrowhead configurations. The S_a_ value dictates the flow direction within the pillar array. Within Fig. 4E–H, dashed boxes highlight the interfaces between fluids with distinct concentrations. These interfaces play a crucial role in species diffusion and mixing in the channel. Red arrows indicate the flow direction through the pillar array. When S_a_ has small values, the proximity of the pillar columns together promotes the flow in the direction alongside the pillar columns (Fig. 4E,G). This causes the flow to go alongside the pillar column and then get back to the center of the channel. In this process, the analytes get distributed through the pillar rows. Consequently, the interface between fluids with different concentrations gets separated by pillars throughout the channel and recombines, enhancing the mixing index. Conversely, for larger S_a_ values, the distance between pillar columns is sufficient to balance the resistance in both horizontal and column-aligned directions (Fig. 4F,H). This results in fluid flow occurring predominantly horizontal, reducing the distribution of the analytes through the pillars and separation of the interface between the two fluids leading to a lower mixing index. To be noted that in case of perfect mixing, the color in the mixing box at the end of the channel becomes green with relative concentration of 0.5 (Fig. 4A–D).

**Figure 5:**
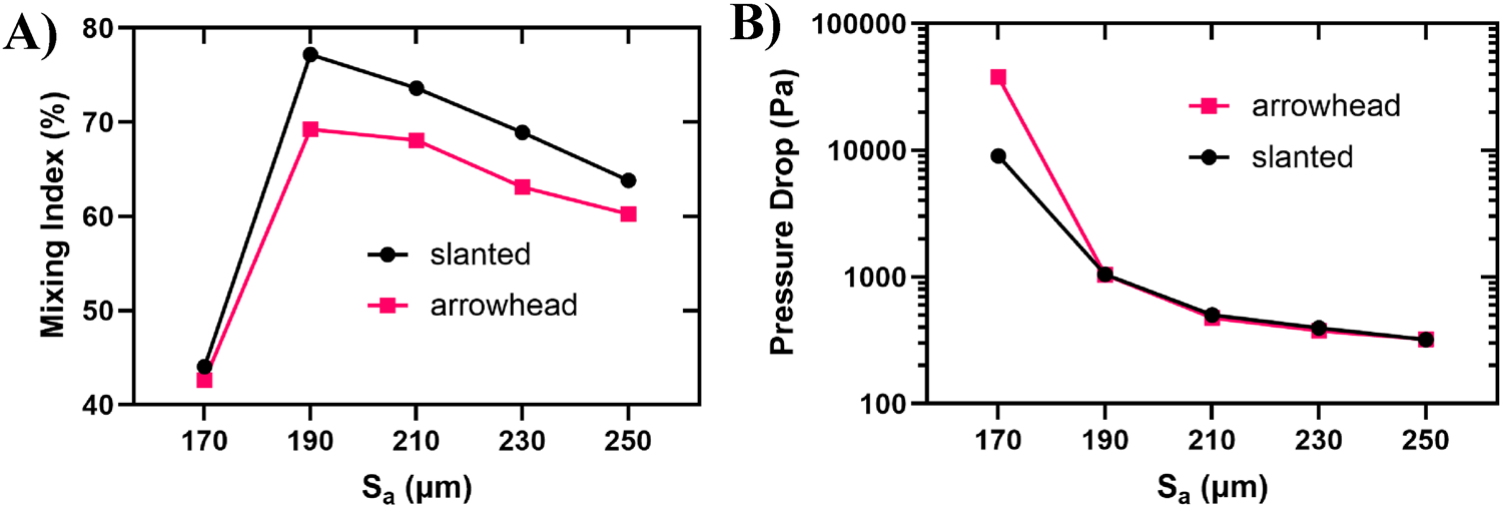
Mixing index and pressure drop variations with S_a_. (**A**) Mixing index and (**B**) pressure drop.

Figure 5A illustrates the quantified comparison of mixing indices between the two configurations concerning S_a_. The mixing index in the arrowhead configuration is marginally lower than that in the slanted configuration. This discrepancy can be attributed to the flow direction of the two fluids with different concentrations. In the arrowhead configuration (see Fig. 4D), the red fluid descends alongside the column of pillars, while the blue fluid ascends. This results in less effective contact between the fluids compared to the slanted configuration. In the slanted configuration (see Fig. 4B), the red fluid ascends alongside the row of pillars, effectively mixing with the blue fluid. This elongates the interface between the two fluids, consequently enhancing the mixing index. To be noted that in all sections of this study, the dye concentrations are illustrated and mixing indices are computed once the mixing process reaches the steady state at the end of the channel. Based on Fig. 5A, in both configurations, the mixing index for the case with $S_{a} = 170\;\upmu\text{m}$ is the lowest value with relatively high difference in comparison to other cases. This is because in this case, the columns of pillars are tightly packed together in a way that the transport of analytes gets too hard leading to a low mixing efficiency. Regarding the pressure drop in Fig. 5B, the highest value is attributed to the $S_{a} = 170\;\upmu\text{m}$, because of the high resistance of the tightly-packed pillars.

Pressure drop is a crucial parameter in the design of micromixers. While incorporating obstacles can enhance mixing efficiency, it also has the potential to elevate the pressure drop. A higher pressure drop necessitates increased pressure and driving force to drive fluids through the channel. This heightened pressure may lead to the microfluidic chip bursting or rupture of the bonding of the chip. In Fig. 5B, the pressure drop (difference in pressure between the inlet and outlet) for both configurations is depicted in relation to S_a_. As S_a_ increases, the distance between pillar columns widens. Consequently, fluid can traverse through the pillars more effortlessly, resulting in a decrease in the pressure drop.

### Effect of gap size (G)

**Figure 6:**
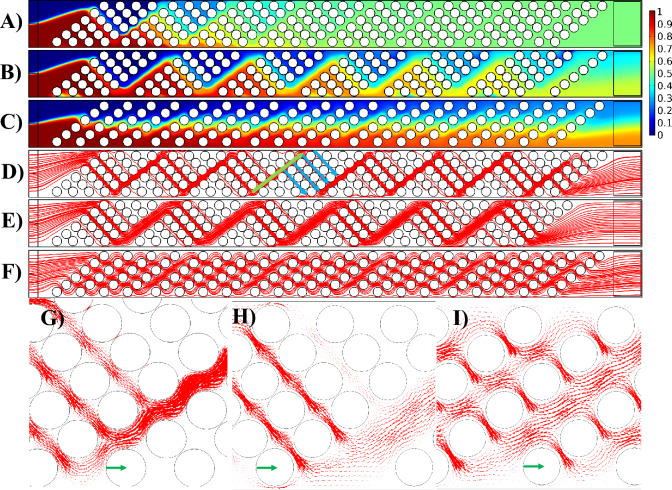
Demonstration of the flow pattern and mixing efficiency with respect to gap size (G) in the slanted configuration. Dye concentration throughout the channel with (**A**) $S_{a} = 170 \;\upmu\text{m}$ and $G = 50 \;\upmu\text{m}$, (**B**) $S_{a} = 170 \;\upmu\text{m}$ and $G = 200\;\upmu{\text{m}}$, and (**C**) $S_{a} = 250 \;\upmu\text{m}$ and $G = 50 \;\upmu\text{m}$. Streamlines passing through the pillars with (**D**) $S_{a} = 170\;\upmu{\text{m}}$ and $G = 50 \;\upmu\text{m}$, (**E**) $S_{a} = 170\;\upmu{\text{m}}$ and $G = 200 \;\upmu\text{m}$, and (**F**) $S_{a} = 250 \;\upmu\text{m}$ and $G = 50 \;\upmu\text{m}$. Magnified view of the velocity vectors through the pillars for (**G**) $S_{a} = 170\;\upmu{\text{m}}$ and $G = 50 \;\upmu\text{m}$, (**H**) $S_{a} = 170 \;\upmu\text{m}$ and $G = 200 \;\upmu\text{m}$, and (**I**) $S_{a} = 250\; \upmu\text{m}$ and $G = 50 \;\upmu\text{m}$. The reference vectors (green) show 0.05 m/s.

In this section, the impact of the gap size (G) between groups of pillars, with a fixed pillar diameter of $100 \;\upmu\text{m}$ is explored. Figure 6 illustrates the mixing efficiency and flow characteristics within the channel, emphasizing the role of the gap as an analyte distributor (see Fig. 6D). In the slanted configuration, the red fluid ascends before each pillar group (green arrow in Fig. 6D), distributing itself among various pillar rows (blue arrows). This distribution enhances the diffusion between fluids with distinct concentrations, elongating the interface and thereby increasing the mixing index. Comparing Fig. 6A,B highlights the effect of gap size (G) while keeping S_a_ constant. The comparison reveals that an increase in G results in decreased mixing efficiency. This is attributed to the diminishing resistance in the gap region (green arrow in Fig. 6D) as G increases, causing uneven distribution among the rows of pillars (blue arrows). In simpler terms, less resistance in the gap area leads to the fluid choosing to go up in that area (green arrow) instead of spreading out through the pillar rows (blue arrows). This causes a significant amount of the fluid to go up and pass through the final rows of pillars (upper blue arrows). This uneven distribution is evident in Fig. 6A,B, where the dye distribution is more uniform in Fig. 6A across the pillar rows compared to Fig. 6B. A comparison of Fig. 6G,H reveals another contributing factor: the smaller gap size acts as a sinusoidal channel, which promotes mixing. However, as the gap size increases, the gap region behaves more like a straight channel, having little to no effect on the mixing of analytes. Furthermore, the degree of contraction and expansion in the gap region can have an effect on the mixing efficiency. Figures 6G,H reveals that smaller gap sizes result in more pronounced contractions and expansions in the gap region, contributing to better mixing. In fact, contraction–expansion regions enhance mixing efficiency by creating shear and elongation effects. As fluid passes through the narrow contraction region, it accelerates, increasing shear rates and stretching fluid elements. Upon entering the expansion region, the fluid decelerates and spreads out, causing the layers to fold over one another. These alternating high and low shear regions disrupt the orderly flow, promoting interfacial contact between fluid streams and enhancing diffusion, thereby significantly improving mixing efficiency even in the absence of turbulence. Figure 8A provides a quantified comparison of the mixing index across various S_a_ and G values for the slanted configuration.

The comparison between Fig. 6A,C highlights the influence of gap size (G) under different S_a_ values. The dye distribution illustrates that, with a constant gap size, increasing S_a_ results in a decrease in the mixing efficiency. Figure 6D,F demonstrate that as S_a_ increases, the distance between pillars increases. As a result, fluid can easily flow horizontally through the pillars due to low resistance, and there is not much upward flow in the gap region in Fig. 6F compared to Fig. 6D (green arrow). Consequently, there is reduced distribution of the fluid among various rows of pillars (blue arrows), leading to a smaller interface for diffusion. In Fig. 6I, a magnified view of velocity vectors for $S_{a} = 250 \;\upmu\text{m}$ and $G = 50 \;\upmu\text{m}$ is presented, clearly depicting that the fluid can effortlessly traverse through the pillars without significant alteration in its direction alongside the pillar columns.

Figure 8A illustrates that the slanted configuration with no gap ($G=0$) provides the lowest mixing index across all S_a_ values. In the slanted configuration, as the pillar columns slope downward, the fluids naturally flow in a downward direction. Introducing a gap between groups of pillars reverses this flow, causing the fluid to move upward in the gap and then distributing it downward into the next pillar group. This upward and downward movement significantly increases the mixing interface. In the absence of a gap, as seen in the gap-less system, there is no upward flow in the gap and subsequent distribution, resulting in a lower mixing index (compare Figs. 4A and 6A).

Figure 8C illustrates the pressure drop for the slanted configuration at various S_a_ values with respect to G. As anticipated, an increase in the gap size leads to a reduction in the pressure drop. This is attributed to the lower resistance against the flow, given the increased free space in the channel and a decreased number of pillars.

**Figure 7:**
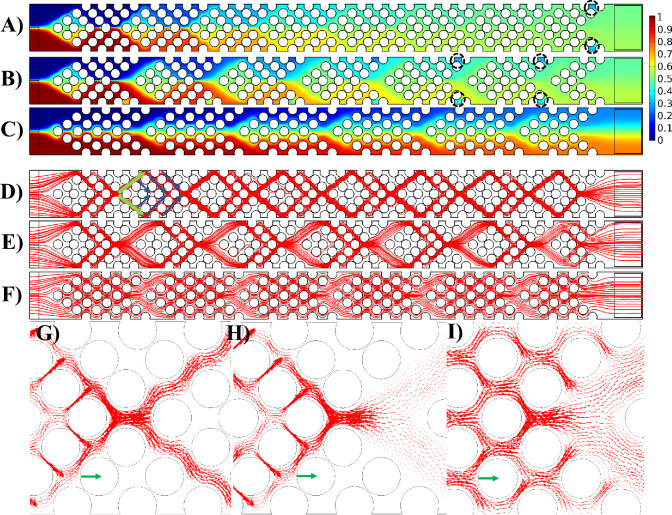
Demonstration of the flow pattern and mixing efficiency with respect to gap size (G) in the arrowhead configuration. Dye concentration throughout the channel with (**A**) $S_{a} = 170\;\upmu{\text{m}}$ and $G = 50\;\upmu{\text{m}}$, (**B**) $S_{a} = 170\;\upmu{\text{m}}$ and $G = 200\;\upmu{\text{m}}$, and (**C**) $S_{a} = 250 \;\upmu\text{m}$ and $G = 50\;\upmu{\text{m}}$. Streamlines passing through the pillars with (**D**) $S_{a} = 170 \;\upmu\text{m}$ and $G = 50 \;\upmu\text{m}$, (**E**) $S_{a} = 170\;\upmu{\text{m}}$ and $G = 200 \;\upmu\text{m}$, and (**F**) $S_{a} = 250 \;\upmu\text{m}$ and $G = 50 \;\mu m$. Magnified view of the velocity vectors through the pillars for (**G**) $S_{a} = 170 \;\upmu\text{m}$ and $G = 50 \;\upmu\text{m}$, (**H**) $S_{a} = 170 \;\upmu\text{m}$ and $G = 200\;\upmu{\text{m}}$, and (**I**) $S_{a} = 250 \;\upmu\text{m}$ and $G = 50 \;\upmu\text{m}$. The reference vectors (green) show 0.05 m/s.

**Figure 8:**
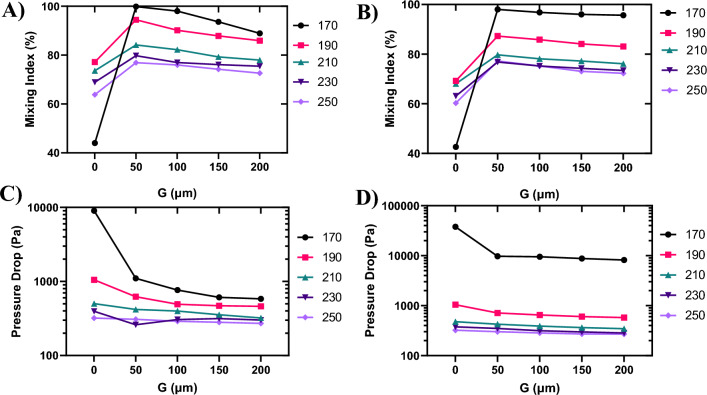
Mixing index and pressure drop variations with the gap size (G). Mixing index in (**A**) the slanted configuration and (**B**) the arrowhead configuration. Pressure drop in (**C**) the slanted configuration and (**D**) the arrowhead configuration.

In the arrowhead configuration, a similar pattern in the variations of the mixing index and pressure drop is observed. Figure 7 illustrates the mixing efficiency and flow characteristics within the channel, emphasizing the role of the gap G as an analyte distributor (see Fig. 7D) in the arrowhead configuration. In this configuration, the interface between the red and blue fluids becomes separated, with part ascending and part descending before each pillar group (indicated by green arrows in Fig. 7D). Subsequently, these separated flows are distributed through the pillar rows (depicted by blue arrows in Fig. 7D). After this distribution, the separated flows come into contact again after the pillar group and are once more separated in the next gap. This distribution enhances diffusion between fluids with distinct concentrations, elongating the interface and consequently increasing the mixing index. Comparing Fig. 7A,B underscores the impact of gap size (G) while maintaining a constant S_a_. The comparison reveals that an increase in G leads to decreased mixing efficiency. This is attributed to the diminishing resistance in the gap region as G increases, causing uneven distribution among the row of pillars. In simpler terms, the decreased resistance in the gap region causes the fluid to prefer flowing along the column of pillars in this area (green arrows) instead of distributing through the pillar rows (blue arrows). As a result, a substantial portion of the fluid moves along the pillar rows in the gap and passes through the last rows of pillars (blue arrows near the upper and lower walls of the channel). This uneven distribution is evident in Fig. 7A,B, where the dye distribution is more uniform in Fig. 7A across the pillar rows compared to Fig. 7B. Another contributing factor is the degree of contraction and expansion in the gap region. A comparison of Fig. 7G,H reveals that smaller gap sizes result in more pronounced contractions and expansions in the gap region, contributing to better mixing. Additionally, Fig. 6G,H reveals another contributing factor: the smaller gap size functions as a sinusoidal channel, enhancing mixing. Conversely, as the gap size increases, the gap region behaves more like a straight channel, reducing its effect on analyte mixing. Figure 8B provides a quantified comparison of the mixing index across various S_a_ and G values for the arrowhead configuration.

The comparison between Fig. 7A,C sheds light on the impact of gap size (G) under different S_a_ values in the arrowhead configuration. The dye distribution reveals that, with a constant gap size, an increase in S_a_ results in a decrease in mixing efficiency. Figure 7D,F demonstrate that as S_a_ increases, the distance between pillars widens. Consequently, the fluid can easily flow horizontally through the pillars due to low resistance, resulting in minimal upward and downward flow in the gap region in Fig. 7F compared to Fig. 7D (indicated by green arrows). Consequently, there is diminished distribution of the fluid among various rows of pillars (depicted by blue arrows), leading to a smaller interface for diffusion. In Fig. 7I, a magnified view of velocity vectors for $S_{a} = 250 \;\upmu\text{m}$ and $G = 50 \;\upmu\text{m}$ is presented, clearly depicting that the fluid can effortlessly traverse through the pillars without significant alteration in its direction alongside the pillar rows.

An important observation from Fig. 7A,B (indicated by dashed circles) is the presence of elevated concentrations of blue fluid in these areas. This can be attributed to the initial condition assumption, where it was presumed that all domains except the inlet of the red fluid (Fig. 1A) were filled with the blue fluid. Due to the proximity of these regions to the walls with no-slip boundary conditions and the narrow spaces between the pillars, the red fluid struggles to adequately mix with the initial blue fluid, resulting in a sustained high concentration of the blue fluid in these regions.

Figure 8B illustrates that the arrowhead configuration with no gap ($G=0$) yields the lowest mixing index across all S_a_ values. In the arrowhead configuration, the pillar columns slope downward in the upper half of the channel and slope upward in the lower half. Consequently, the fluids naturally converge toward the middle of the channel, resulting in a short interface between the two fluids solely in the middle of the channel, leading to a low mixing efficiency (Fig. 4C). Introducing a gap between groups of pillars reverses this flow, causing the fluid to move upward and downward toward the walls of the channel (green arrows in Fig. 7D) and then distributing it into the next pillar group toward the middle of the channel (blue arrows in Fig. 7D). This upward and downward movement significantly increases the mixing interface. In the absence of a gap, as observed in the gap-less system, there is no upward and downward flow in the gap and subsequent distribution, resulting in a lower mixing index (compare Figs. 4C and 7A).

Figure 8D illustrates the pressure drop for the arrowhead configuration at various S_a_ values with respect to G. As anticipated, an increase in the gap size leads to a reduction in the pressure drop. This is attributed to the lower resistance against the flow, given the increased free space in the channel and a decreased number of pillars.

Based on Fig. 8A,B, in both configurations, the mixing indices for the cases with $G=0$ are the lowest values with relatively high differences in comparison to other cases. This is because in these cases, the columns of pillars are tightly packed together without any gap in the channel in a way that the transport of analytes gets too hard leading to a low mixing efficiency. Regarding the pressure drop in Fig. 8C,D, the highest values are attributed to the cases with $S_{a} = 170 \;\upmu\text{m}$ and $G=0$, because of the high resistance of the tightly-packed pillars.

### Effect of pillar diameter

**Figure 9:**
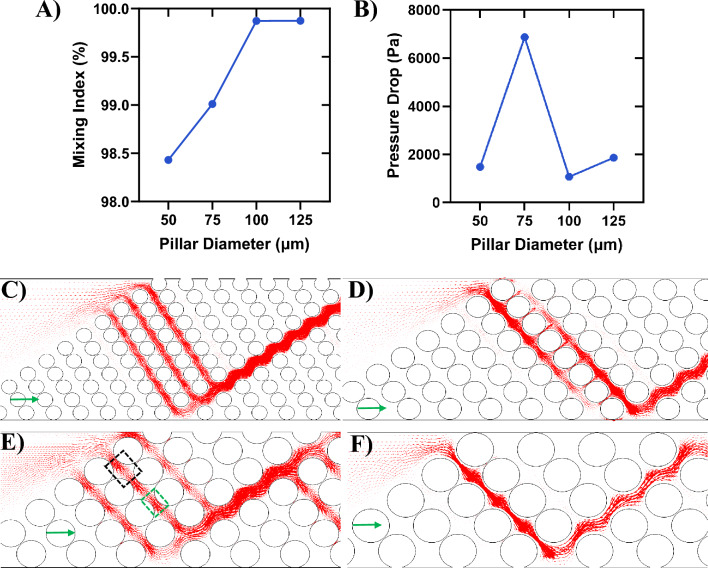
Mixing index and pressure drop variations with the pillar diameter (D_p_). (**A**) Mixing index and (**B**) pressure drop. The open passways between the row of pillars for the pillar diameter of (**C**) $D_{p} = 50 \;\upmu\text{m}$, (**D**) $D_{p} = 75\;\upmu{\text{m}}$, (**E**) $D_{p} = 100 \;\upmu\text{m}$, and (**F**) $D_{p} = 125 \;\upmu\text{m}$. The reference vectors (green) show 0.1 m/s.

**Figure 10:**
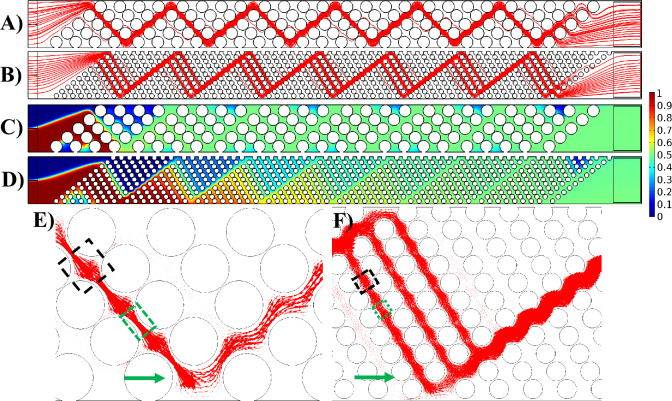
Demonstration of the flow pattern and mixing efficiency with respect to the pillar diameter (D_p_) in the slanted configuration. Streamlines passing through the pillars for (**A**) $D_{p} = 125\;\upmu{\text{m}}$ and (**B**) $D_{p} = 50 \;\upmu\text{m}$. Dye concentration for (**C**) $D_{p} = 125\;\upmu{\text{m}}$ and (**D**) $D_{p} = 50\;\upmu{\text{m}}$. Magnified view of the velocity vectors through the pillars for (**E**) $D_{p} = 125\;\upmu{\text{m}}$ and (**F**) $D_{p} = 50 \;\upmu\text{m}$. The reference vectors (green) show 0.1 m/s.

In this section, the impact of pillar diameter on both the mixing index and pressure drop is examined. Building upon insights gained from previous sections, where the pillar array with $S_{a} = 170 \;\upmu\text{m}$ and $G = 50 \;\upmu\text{m}$ exhibited the highest mixing index, these parameters were selected for further investigation into pillar diameter effects. To ensure a fair comparison across different pillar diameters and isolate the impact of diameter variation, the distances between pillars within each column and between adjacent columns of pillars were maintained constant. This investigation is conducted within the slanted configuration, with $S_{a} = 170 \;\upmu\text{m}$ and $G = 50 \;\upmu\text{m}$. Figure 9A displays the variations in mixing index with respect to pillar diameter. As the pillar diameter increases from $50$ to $125\;\upmu{\text{m}}$, the mixing index increases, as well (compare Fig. 10C,D). This phenomenon can be attributed to the larger pillars providing more space within the passages between pillar rows, for a constant distance between pillars in each column and between the adjacent columns. A comparison between Fig. 10E,F reveals that with $D_{p} = 125\;\upmu{\text{m}}$, there is greater expansion between pillars (indicated by the black dashed box) compared to $D_{p} = 50 \;\upmu\text{m}$. To have a fare comparison, the distances between pillar rows (indicated by the green boxes in Fig. 10E and F) remain constant across cases with different diameters. As a result, the size disparity between contraction (green dashed box) and expansion (black dashed box) increases with larger pillar diameter, leading to improved mixing and higher mixing index values. Furthermore, in the cases with smaller pillar diameters, the overall space between rows of pillars (sum of contraction and expansions) is reduced, resulting in higher flow velocity and shorter residence time for analytes to mix (compare Fig. 10A,B). Consequently, this contributes to a lower mixing index. To be noted that the number of pillar columns in each pillar group changes with pillar diameter to have a constant pillar group length.

An important observation from Fig. 10C,D is the unmixed areas near upper and lower walls of the channel. This can be attributed to the initial condition assumption, where it was presumed that all domains except the inlet of the red fluid (Fig. 1A) were filled with the blue fluid. Due to the proximity of these regions to the walls with no-slip boundary conditions and the narrow spaces between the pillars, the red fluid struggles to adequately mix with the initial blue fluid, resulting in a sustained high concentration of the blue fluid in these regions.

Figure 9B presents the variations in pressure drop with pillar diameter. The pressure drop depends on the number and width of the open passages between the rows of pillars (see Fig. 9C–F). The distance between the rows of pillars remains constant for all cases (green dashed box in Fig. 9E). As the pillar diameter increases, the overall space between the rows of pillars also increases due to the larger gaps between individual pillars (black dashed box in Fig. 9E). This results in each passage having lower resistance to flow as the pillar diameter increases. The micromixers with pillar diameters of 50 μm and 100 μm exhibit the lowest pressure drops because they each have three open passages (Fig. 9C,E). The micromixer with a 100 μm pillar diameter experiences the lowest pressure drop due to the wider passages, which reduces overall resistance. Following these, the micromixer with a 125 μm pillar diameter has a lower pressure drop because it features one open passage (Fig. 9F). The highest pressure drop occurs with the 75 μm pillar diameter case. In this case, there is one passage, but it is blocked by the lower wall of the channel, forcing the flow to move through the tight spaces between pillars (Fig. 9B), resulting in a relatively high pressure drop.

Notably, the pillar array with a diameter of $100 \;\upmu\text{m}$ exhibits the lowest pressure drop while achieving the second highest mixing index (Fig. 9A). Comparing our findings with similar studies conducted by other researchers, our results demonstrate a reasonable pressure drop alongside a considerable improvement in the mixing index. For instance, in the work by Xia et al.^68^, they employed micromixers featuring contraction–expansion and obstacles to enhance mixing. In their study, at $Re=1$, the pressure drop was approximately $1000 Pa$, with a mixing index of about 48%. In contrast, in our study, the pressure drop was maintained around $1000 Pa$ while the mixing index was significantly increased to more than 98% (Fig. 9A). This suggests that our approach yields superior mixing efficiency without substantially increasing the pressure drop, which is a noteworthy advancement in microfluidic micromixer design.

### Effect of vertical shift (S_y_) of pillar groups

**Figure 11:**
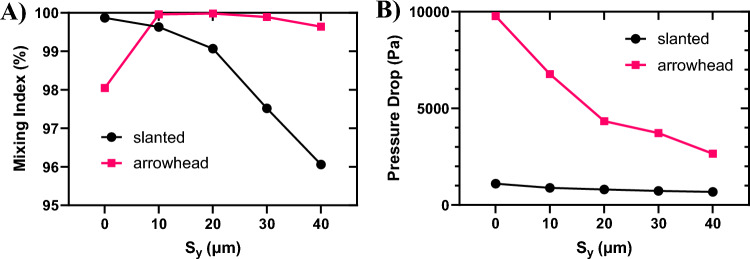
Mixing index and pressure drop variations with the vertical shift (S_y_). (**A**) Mixing index and (**B**) pressure drop.

One of the factors influencing the mixing efficiency in this channel is the vertical shift (S_y_) between the groups of pillars. Based on the results from previous sections, the best case in terms of mixing index and pressure drop with $S_{a} = 170 \;\upmu\text{m}$, $G = 50 \;\upmu\text{m}$, and $D_{p} = 100\;\upmu{\text{m}}$ was chosen. Having a pillar array with these parameters, S_y_ was changed from $0$ to $40 \;\upmu\text{m}$ and the variations in the mixing index and pressure drop was investigated. As illustrated in Fig. 11A, in the slanted configuration, increasing S_y_ from $0$ to $40 \;\upmu\text{m}$ causes a 4% decrease in the mixing index. Conversely, in the arrowhead configuration, augmenting S_y_ initially leads to an increase in the mixing index, followed by a slight decline. The maximum mixing index for this configuration is achieved at $S_{y} = 10\;\upmu{\text{m}}$.

The changes in pressure drop with S_y_ are illustrated in Fig. 11B. As the vertical shift increases, the pressure drop decreases. This is due to the fact that with an increasing vertical shift, the rows of pillars in adjacent groups are not directly aligned with each other (see Fig. 1B). Consequently, this reduces the resistance to flow, resulting in a decrease in the pressure drop.

### Changes in mixing index over time and channel length

Based on the parameters investigated and the results obtained in the previous sections, the optimal micromixers in terms of mixing index are as follows: slanted configuration with $S_{a} = 170 \;\upmu\text{m}$, $G = 50 \;\upmu\text{m}$, $D_{p} = 100 \;\upmu\text{m}$, and $S_{y} = 0\;\upmu{\text{m}}$; and arrowhead configuration with $S_{a} = 170\; \upmu\text{m}$, $G = 50 \;\upmu\text{m}$, $D_{p} = 100 \;\upmu\text{m}$, and $S_{y} = 10 \;\upmu\text{m}$ (Fig. 11A). Now, for these micromixers, the variations in the mixing index over time until reaching steady state at the end of the channel, within the mixing box, is examined (Fig. 1A). Additionally, exploring the mixing index variations along the channel from the inlet to the outlet provides valuable insight into the mixing length.

**Figure 12:**
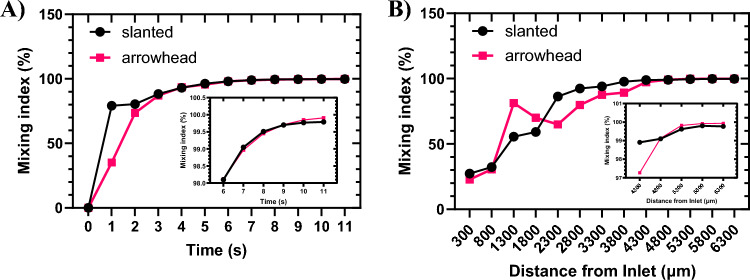
Mixing index variations with time and distance from inlets. (**A**) Time-dependent variations of mixing index until reaching steady-state. (**B**) Mixing index variations at different positions along the channel at the steady state.

Figure 12A illustrates the changes in mixing index over time. The mixing index is calculated within the mixing box at the end of the channel. As time progresses, the mixing index increases until the mixing process reaches a steady state. By the 8-s mark, a steady state at the end of the channel with a mixing index of 99.5% is achieved. In the steady state, both the slanted and arrowhead configurations exhibit high mixing indices near 100%, which is a significant achievement. By the 3-s mark, both configurations can attain mixing indices exceeding 90%.

Another crucial parameter in micromixers is the mixing length, which determines the overall length of the channel over which perfect mixing can be achieved. Figure 12B demonstrates the mixing index variations in different positions along the channel. In both configurations, mixing indices higher than 99% can be achieved with a length of $4300\;\upmu{\text{m}}$. In similar studies, such as that by Xia et al.^68^, for Re around 1, the reported mixing index is approximately 48% with a channel length of $1600 \;\upmu\text{m}$. In our study, the mixing indices at a distance of $1600 \;\upmu\text{m}$ from the inlet are 54% and 75% for the slanted and arrowhead configurations, respectively.

As a result, both the slanted and arrowhead configurations with the optimum parameters introduced in this section represent ideal micromixers, offering high mixing indices in a short time and with a short mixing length. It is important to note that micromixers typically achieve high mixing indices close to 100% at extremely low ($Re\ll 1$) or high Reynolds Numbers ($Re>40$)^68^. This phenomenon occurs because at low Re, the residence time increases, allowing analytes more time to mix. Conversely, at high Re, separated vortices can form, enhancing mixing efficiency. However, at moderate Re, the residence time is shorter compared to low Re, and the absence of separated vortices like those at high Re results in poor diffusion and convection, leading to lower mixing efficiency. However, the micromixers introduced in this study demonstrate mixing indices higher than 99.7% at $Re\approx 1$.

## Conclusions

This study employed numerical simulations to explore the fluid dynamics and mass transfer characteristics of two innovative micromixers featuring pillar arrays. Principles such as contraction–expansion and split-recombine were used and the mixing efficiency and pressure drop were studied in different pillar arrays. Introducing two distinct configurations of pillar arrays, slanted and arrowhead, parameters including pillar diameter, gap size between pillar groups, distance between pillars, and vertical shift between pillar groups are examined. Through this comprehensive analysis, micromixers with optimal parameters were identified. Generally, the arrowhead configuration showed higher pressure drop with a slightly higher mixing index at the steady state. Notably, the pressure drop remained minimal at 1102 Pa in the optimal slanted configuration. Both optimal slanted and arrowhead configurations were capable of achieving exceptional mixing efficiency exceeding 99.7% at moderate Reynolds number ($Re = 1$). This is while at this order of Re achieving high mixing index is challenging. Additionally, this study examined variations in mixing index over time and along different positions within the channel. Both configurations exhibited short mixing lengths and times. Both slanted and arrowhead configurations achieved steady state with a 99.5% mixing index at the end of the channel by 8 s. Furthermore, by 3 s, both configurations surpassed 90% mixing indices. At a distance of $4800\;\upmu{\text{m}}$ from the inlet, both configurations achieved mixing indices higher than 99%. At a distance of $1300\;\upmu{\text{m}}$, the mixing indices were 53% and 80% for the slanted and arrowhead configurations, respectively. Overall, the swift mixing, minimal pressure drop, and short mixing lengths observed in these novel micromixers position them as promising options for microfluidic applications.

## Data Availability

The datasets generated and/or analyzed during the current study are not publicly available due to requirement of confidentiality, but are available from the corresponding author on reasonable request.
